# Sensing and Control Strategies for a Synergy-Based, Cable-Driven Exosuit via a Modular Test Bench

**DOI:** 10.3390/s23104713

**Published:** 2023-05-12

**Authors:** Ashwin Jayakumar, Daniel Rodríguez Jorge, Javier Bermejo-García, Rafael Agujetas, Francisco Romero-Sánchez

**Affiliations:** Departamento de Ingeniería Mecánica, Energética y de los Materiales, Escuela de Ingenierías Industriales, Universidad de Extremadura, Avda. de Elvas S/N, 06006 Badajoz, Spain

**Keywords:** exosuit, modular test bench, postural synergies, cable-driven actuation systems, rehabilitation engineering, control, circuit design

## Abstract

Ageing results in the eventual loss of muscle mass and strength, joint problems, and overall slowing of movements, with a greater risk of suffering falls or other such accidents. The use of gait assistance exoskeletons can help in the active aging of this segment of the population. Given the user specificity of the mechanics and control these devices need, the facility used to test different design parameters is indispensable. This work deals with the modeling and construction of a modular test bench and prototype exosuit to test different mounting and control schemes for a cable-driven exoskeleton or exosuit. The test bench allows the experimental implementation of postural or kinematic synergies to assist multiple joints by using only one actuator and the optimization of the control scheme to better adapt to the characteristics of the specific patient. The design is open to the research community and it is expected to improve the design of cable-driven systems for exosuits.

## 1. Introduction

The United Nations predicts that the population above the age of 65 will increase from 9% to 16% by the year 2050 [1]. The field of soft assistive exoskeletons, otherwise known as ‘exosuits’, is a relatively new development that may be a solution for this group, given its success with patients experiencing other gait-related disabilities, such as post-stroke patients [2,3,4,5]. One of the first works to describe the use of exosuits for lower limb gait assistance was References [6,7], which analyzed the use of textiles for force transmission. This exosuit actuated the hip and ankle, with pressure sensors at the heel to detect the gait phase. The system stored the last five steps to determine the gait period. It used position control based on a predetermined gait curve, which was sent as an input for the motor to follow. The design achieved a metabolic cost reduction of 6.4%.

A multi-joint external actuator used to test iterative control using IMUs in a lower limb exosuit is shown in [8]. It has the actuation system mounted on a table next to the user in order to perform the tests, with Bowden cables transmitting the forces to the subject. The controller uses the actuator position and force as feedback to control the hip extension. A similar setup was used in [9], but this time with an ankle plantar flexion actuation included.

Regarding upper limb exosuits, one particularly interesting design proposed in [10,11] describes a wearable exosuit based on postural synergies. The design consisted of one motor that actuated a pulley train with the aim of helping to grasp objects with the fingers of that hand. The diameter of each pulley was determined by synergies, allowing the system to actuate various fingers with just one motor. This design used clutches to halt the system and hold the fingers at a given position without the need to start the motor. Of special interest is Reference [9], which describes the use of a test bench to test the backlash in Bowden cables. The test bench consisted of a motor with an encoder, a secondary encoder to measure cable displacement, input and output pulleys, springs, and the Bowden cable itself. The setup was controlled via a Quanser QUARC controlled via MATLAB. This test bench was useful for analyzing different control configurations. Another test bench was used in [12]; it involved an actuator consisting of a spool, motor, bearings, etc., connected to a holder via Bowden cables, which had a force sensor, spring, etc. This setup was used to test the actuator impedance bandwidth based on the applied torque and measured output position. Xu et al. [13] analyzed the mechanical implementation of synergies as planetary gears. This system transforms two rotating inputs into 13 rotating outputs for each hand segment via a specifically defined pulley system. To test the performance of the upper limb exoskeleton with shape-memory alloys, Copaci et al. [14] used a test bench that could simulate a forearm with 1-DOF. It used a four-term bilinear PID controller to achieve antagonistic control.

To test Bowden cables for the transmission of forces in exoskeletons, [15] designed two different test setups (consisting of a motor, pulley, and Bowden cable, each mounted on a test bench) by running the cables from a motor in a push–pull configuration in both cases. A dSpace DS1103 was used for control. The first prototype was capable of testing different loads via a load bar. It was proven to be useful for the actuation of a human arm and helped relocate the weight of the motor from the arm. In [16], the authors used an aluminum frame with steel parts, load cells, and Arduino Uno, among others, to evaluate upper limb exoskeletons. This setup provided a simple and repeatable option for testing and yielded good results for evaluating the performances of different configurations for upper limb exoskeletons. Going further, in the work by Nguyen et al. [17], the test bench was set up on a metal frame with all of the major components, such as loading springs, pulleys, torque sensors, servomotor, etc., mounted on it. They tested output torque tracking methods to control the output torque of a servo drive in a system with 1-DOF. Their scheme, which used a Bouc–Wen hysteresis model, provided improved results, as shown by the tests conducted in their setup compared to other options.

An important fact is that the assistive force produced needs to significantly offset the parasitic losses due to the added weight of the overall system, the transmission losses, etc., for an exosuit to ultimately be useful in gait assistance. This was apparent in [18], where a total system weight of 9.1 kg meant that the system was unable to provide useful gait assistance to the wearer.

The reduction of weight in gait-assist devices is not an easy task. The synergies concept is often used to characterize the greatest number of possible movements with the least number of actuators [19,20]. Synergies provide a way to correlate the actuation of each segment with the number of actuators available. This can be used to combine multiple actuators for gait assistance or to reduce the number of actuators needed. This article uses the latter to reduce the number of actuators needed to provide assistance. Synergies can help minimize the costs and complexity in the overall design of an exosuit, as fewer actuators mean that fewer auxiliary components are required in the system, which significantly reduces the final cost of the prototype.

In order to characterize these synergies, both the mathematical model and the real-world prototype are important to verify the functioning of the system. To decide on an actuation method that will serve well in the final exosuit design, it is necessary to perform several tests while modifying various design parameters to determine which combination is the most optimal for the given project in order to provide appropriate gait assistance to the user at just the right time. Testing such actuation strategies is commonly done in a specialized test bench with the necessary sensing and anchoring defined for the problem at hand, as described in [12,21]. However, these designs are usually very specific to the particular test to be conducted and cannot be used for conducting different experiments without significant modifications. Thus, to overcome these limitations, this project presents the design of a modular test bench that can be used to test different types of actuation systems (motors), control strategies, and configurations (only ankle, only knee, only hip, or any combination henceforth), using modules that can be reused for different tests, allowing for easy mounting to facilitate rapid prototyping. Such a test bench allows one to perform experiments by varying specific parameters, such as the cable type, anchor points, etc., between experiments. Being able to control these variables also allows various tests to be performed with a high degree of repeatability between different tests, which is important when quantifying the effects they produce.

## 2. Materials and Methods

### 2.1. Design Criteria and Concepts

**Figure 1:**
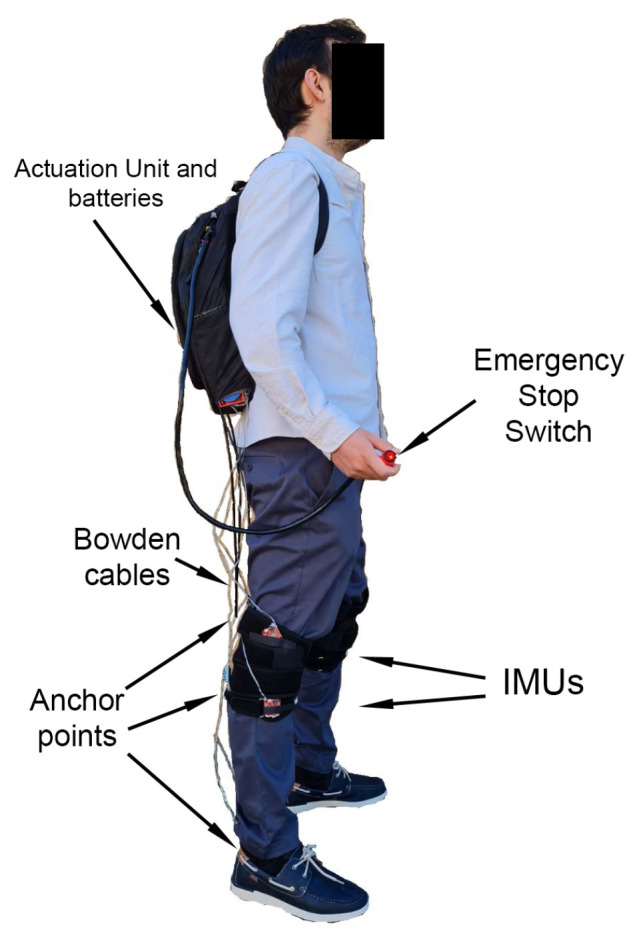
Prototype exosuit based on synergies.

The basis for designing the exosuit was to use the torque curves of each leg segment and attempt to provide a fraction of the aforementioned torque at each segment. The torque curves were calculated from an inverse dynamics model [20] that received its kinematic input from publicly available human gait databases [22]; anthropometric data were estimated from [23] with the available information. The model estimated the motor torque required to assist 100% of the gait cycle for each segment; the final goal was to provide assistance (by about 30%) once the exosuit design was proposed. This restriction was due to the inherent mechanical limitations of cable-driven exosuits, which were described by certain authors based on other state-of-the-art lower-limb, soft-assistive devices, as in [7]. The output was estimated for the shaft of the motor gearbox, before the other transmission elements, such as gears and clutches. In [24], the authors proposed a synergy-based design for gait-assistance exosuits that allowed a single actuator to provide torque at all three lower-limb joints, i.e., hip, knee, and ankle, in both legs. Figure 1 shows a prototype of the proposed exosuit. As shown, the exosuit consists of a backpack where the entire actuation unit is enclosed, providing the necessary actuation via cables to each joint through the anchor points. In order to quantify its performance in a safe environment, the modular test bench is used to prove that the synergy-based design approach is verified and, consequently, the exosuit too. Analyzing a population sample of ten subjects, excellent results were obtained with a single actuator, with strong similarities in the cable extensions during the gait for all joints. This allowed the authors to significantly reduce the weight and price of the overall device, while also providing preliminary design criteria, including pulley radii, required power, etc. Actuating all joints at the same time with just one motor may introduce power and/or torque-related issues that may inhibit the actuation. Thus, the assumption is made that the exosuit will only assist at certain points in the gait cycle; the objective is to predict at which phases the gait assistance can be provided to the subject with the required torque.

Once the required torque at the motor shaft is predicted, the maximum torque required from the motor to actuate the joints happens to be positive at certain points in the gait. Since cables can only transmit force under tension, actuation is restricted to flexion only, which provides the first criterion to determine the actuation phases.

Consequently, if all three joints are mechanically coupled, all joints could only be actuated during a small portion of the gait. Still, each user may require different actuation schemes, focusing on one joint or another, as highlighted in [25] for older adults. To avoid overloading the motor/clutches, and considering the available torque, it was decided to decouple the knee joint from the others, thus increasing the assistance capabilities of the exosuit. An inverse-dynamics 3D model is used to predict the evolution of the biological joint torques, as better described in [20,24].

To analyze the difference in the quality of gait assistance provided by using two actuators instead of one, a test setup was built consisting of two motors, with different pulleys on each one. One motor had a pulley train with two pulleys whose diameters were determined from the data of a test subject in the database [22] (specifically, subject number 29, whose anthropometric parameters were the closest to the average adult), and the other had a train with three pulleys (although only two were used in the test, taking into account the aforementioned details). The train with two pulleys was the first synergy, which was expected to contribute the most to the gait assist. The second train with three pulleys was the second synergy. The first motor was actuated based on the curve generated from the model, and it controlled the cable extension amount by winding or unwinding the cable as required to generate the synergy. Two tests were conducted with one or two active motors, as detailed below. If the concept of synergies works, the test should reveal that the curves obtained with one single motor and the resultant of two motors together should be extremely similar to the theoretically calculated curve, proving that the three segments of two legs can be actuated using just one single motor.

**Figure 2:**
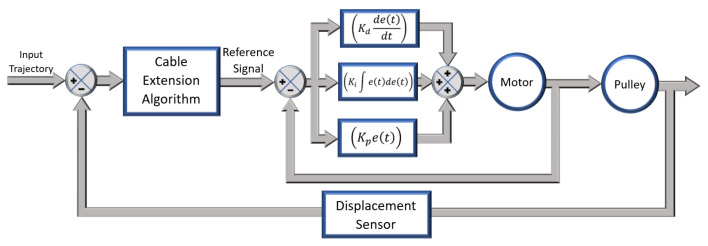
Overall control scheme.

The desired reference trajectory to be followed by each motor was processed and fed to the motor controller. The motors needed to be controlled using a position control algorithm. Since clutches were used to isolate the actuation of the knee from the hip–ankle, the motor was subjected to abrupt changes in the load and needed to respond quickly to these disturbances. A simple proportional or PD controller will not recover fast enough to provide continuous gait assistance without significantly overshooting and drawing more cable than necessary. To eliminate any resulting problems after having engaged the clutch, the position error also needed to be reduced quickly. Thus, in order to account for all of these operational variations, a PID control loop was chosen, as is common for such position control applications [14,21,26,27,28,29]. Importantly, the setup was tuned while taking into account the load attached to the motor, such as pulley trains, etc., to ensure optimal system response. The PID control values were estimated initially by applying the Ziegler–Nichols criteria, which is a heuristic tuning method. The specific tuning rule used was based on ’no overshoot’ parameters [30]. Once the preliminary values of the proportional gain were obtained and the approximate values of the integral and derivative parameters were determined, the system response was tested. In this case, it was decided to iteratively continue adjusting the parameters until a rather aggressive but reasonably precise system response with little to no overshoot and better command tracking was achieved. The overall control scheme is shown in Figure 2.

Two types of input trajectories, i.e., angular position curves and absolute displacement curves, can be used for different tests. To estimate the values needed to control the motor in an angular position, a cable extension estimation algorithm is needed. It needs to take into account the encoder resolution and the absolute reduction of the motor gearbox used. For this, the following expression was derived mathematically:

(1) $$p_{req}=\frac{2^{N_{enc}}\cdot G_{A}\cdot \omega_{req}}{2\cdot \pi \cdot G_{B}}$$

where $p_{req}$ is the position to be sent to the motor, $N_{enc}$ is the number of bits of the encoder attached to the motor, $G_{A}$ and $G_{B}$ are the absolute internal reduction values in the gearbox, and $\omega_{req}$ is the desired angular position. This is the input signal sent to the motor position controller to produce the desired cable displacement needed for the assistive force for the gait.

A similar equation is used for the cable displacement curves where applicable. The resulting cable displacement is measured using a load cell, the encoder, and a linear displacement sensor in cases that require higher precision in absolute resulting displacement. The general control scheme and overall system block diagram remain the same. The absolute error is calculated by comparing the detected position against the input required position.

(2) $$\Delta e_{f}=p_{req}-p_{meas}$$

where $e_{f}$ is the following error, and $p_{req}$ and $p_{meas}$ are the desired input position and encoder reported value, respectively. The displacement as measured by the encoder is calculated by knowing its single-turn resolution in bits ($b_{enc}$), as follows:

(3) $$d_{cable}^{enc}=\frac{n_{pulses}\cdot 2\cdot \pi \cdot r_{pulley}}{2^{b_{enc}}}$$

where $n_{pulses}$ is the number of pulses counted by the encoder, $r_{pulley}$ is the radius of the attached pulley. There are springs used in series to provide a sufficient extension length and a restoring force. The cable tension $T_{cable}$ measured by the load cell is divided by the spring factor of the springs used in series ($K_{total}$) to determine the displacement, $d_{cable}$:

(4) $$d_{cable}=\frac{T_{cable}}{K_{total}}$$

In this case, the springs used had a spring factor of k = 1060 N/m. Each spring had a maximum displacement of 29.7 mm, so three were used in series to achieve a total displacement of up to 90 mm. This range allowed the scale factor for the input curves to be as little as half of the real-world calculated values expected to be used in the exosuit. To measure displacement, a linear displacement sensor connected to the ADC of the Arduino was used, which had a maximum displacement range of 100 mm.

### 2.2. Construction of the Test Bench and Its Modules

To determine the dimensions and tolerances needed, the prototype was modeled in full 3D using CAD software. Each piece was designed and mounted as a virtual assembly to verify cross-compatibility and estimate the spatial position before being mounted on the actual test bench. This helped to design parts with adequate dimensions and tolerances, and to verify compatibility, the number of anchor points, and their positions for cable routing, as well as determine approximate cable lengths, etc., for more complex tests involving multiple components. Once the major elements, such as the supports and pulleys, were designed and dimensioned in CAD, these parts were fabricated by 3D printing using high-resistance PLA 870 and then annealed for use in testing. The motor and pulley holders were designed to accommodate pulley trains of varying dimensions, up to 130 mm in diameter, which allows for the testing of pulley trains of different dimensions without having to fabricate a new holder.

**Figure 3:**
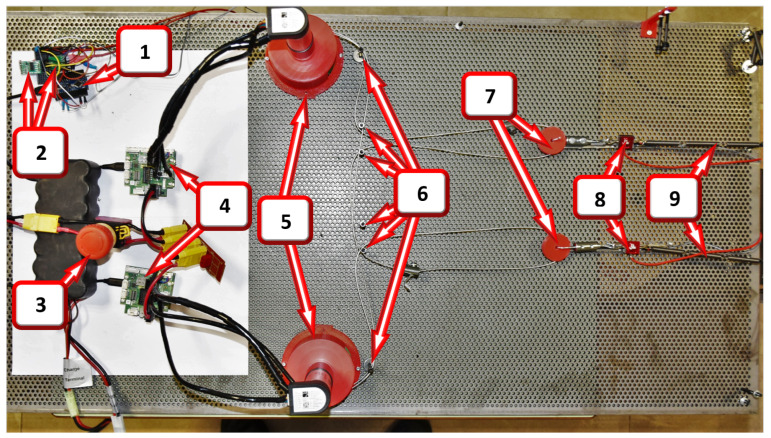
Dual motor synergy test setup: (1) Arduino, (2) load cell amplifiers, (3) emergency stop switch, (4) motor controllers (5) two motors with pulleys and supports, (6) cable guide points, (7) mid pulleys, (8) load cells, and (9) extension springs.

The test setup for testing the synergies is shown in Figure 3, with the Arduino (1) for sensor interfacing, load cell amplifiers (2), emergency stop switch (3), motor controllers (4), and the two motors (5) mounted on their respective supports, each with a specially designed pulley train mounted directly on its axis. The setup also included cable guide points (6) for cable routing, mid pulleys (7) to obtain the output synergy, load cells (8), and extension springs (9).

The test bench itself was made using layers of perforated steel sheets with a consistent profile that facilitated easy modifications of anchor points for the cable that would eventually transmit the forces to the human body in the final exosuit, as well as the addition of the different modules for testing flexibility. The sheets used in this project (R4T6, 1000 mm by 500 mm) had a 6 mm triangular profile with 4 mm holes.

The motors used in the test bench were 200 W Maxon EC 4-pole (BLDC) motors, featuring a maximum torque of 95 mNm. In order to have more torque output, two different gearboxes were used: one with a 33:1 reduction (giving rise to a maximum torque output of 3.13 Nm) and another with a 79:1 reduction ratio (with a torque output of 7.5 Nm). Both motors were equipped with 12/20 absolute multi-turn encoders for feedback in the position control loop. They were connected using a serial synchronous interface (SSI). Maxon EPOS 50/8 CAN modules were used as the motor controllers, in which both the hall sensors and absolute encoders were connected. These controllers could communicate with the PC via USB 2.0 COM or with the Arduino via RS-232, using appropriate signaling hardware and following the object dictionary of the device.

Futek LSB201 S-Beam load cells were implemented to measure tensions and forces while connected to an appropriate amplifier, such as the HX711. They have a maximum load rate of 445 N and can measure both compression and extension. The linear displacement sensor consists of a linear track potentiometer connected to an ADC. It has a 100 mm track length and linearly proportional output, which allows for measuring cable displacements without the need for a high scaling factor. This solution is both economical and easy to implement for the required resolution.

The force transmission elements used were two different types of cables: one being a 1.5 mm steel cable with 6 wires, 7 strands, a maximum load rate of 26 kg, and a safety factor of 5:1. The other was a bicycle brake cable of 1.5 mm with its corresponding brake sheath. For applications where flexibility was a priority, a gear sheath was used. The correct selection of sheaths for the application was important to avoid unnecessary friction and transmission losses.

To interface all of the different sensors used, a 32-bit Arduino DUE was used. The code on the Arduino is designed to receive commands from the host PC script and poll any of the various sensors attached to it as requested by the host. The script on the PC decides which sensors are polled independently based on the needs of the experiment to be performed. This means that the Arduino does not need to be reprogrammed between experiments, only the PC script changes. This Arduino is also used to control the clutch control module if necessary and send commands to the motors. The four-channel clutch control board consists of Panasonic AQV252 solid-state relays and electronics that provide support and protection. Up to four clutches can be connected to this board. This actuation method enables the engagement and disengagement of clutches to control force transmission to the segments of the legs under different operating modes, which are important but beyond the scope of this article. Unlike traditional clutches, these devices do not have mechanical components that wear out, making them last long when operated within specifications.

**Figure 4:**
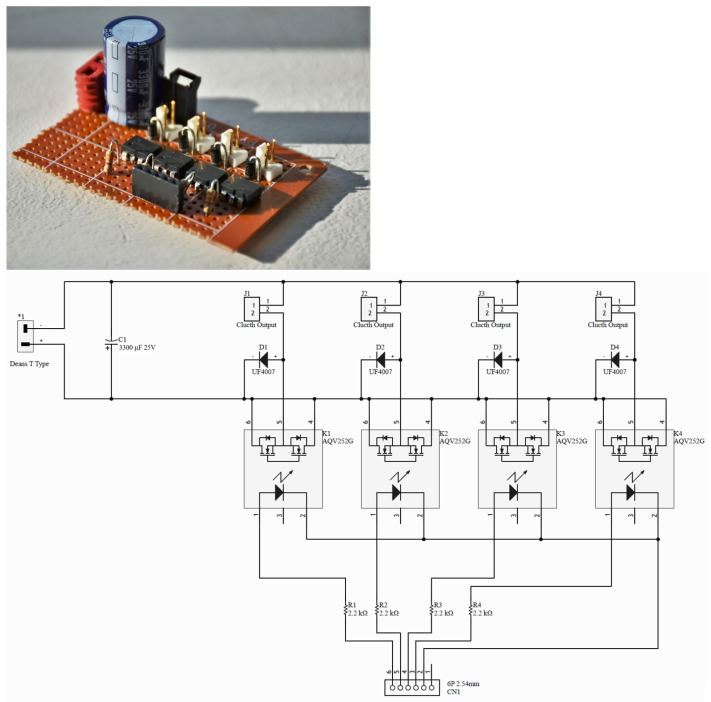
Upper: Four-channel clutch control SSR board. Lower: circuit diagram for the clutch control board. Component Description: 1: Deans T-Type Input Connector to battery. C1: 3300 µF 25 V Capacitor. K1–K4: Panasonic AQV252 SSRs. R1–R4: 2.2 kΩ Resistors. CN1: Control Input Connectors. D1–D4: UF4007 diodes. J1–J4: Deans T-Type Output Connector to EM Clutches.

This module accepts 3.3 V to 5 V input signals at the optically isolated side and can control devices up to 60 V and 2.5 A at the output if used in the bridged mode, as implemented in this system for the actuation of the clutches. The isolation in the SSR prevents the switching noise caused by the inductive flyback from the clutches from affecting the control circuitry. The constructed module is shown in Figure 4. As it is designed with components that can handle significantly more currents and voltages than those encountered in the present application, the board can be reused for experiments that require larger clutches with higher power consumption, without the need to fabricate another board.

With the goal of testing the same hardware to be used in the final exosuit design, a power supply module was designed for voltage regulation and distribution. The exosuit was powered by a six-cell (6S) lithium-polymer battery with a nominal voltage of 22.2 V. The test bench was powered using a 1000 W external benchtop power supply but with current and voltage output limits modeled to closely approximate the characteristics of the aforementioned battery.

The voltage distribution module consisted of three distinct rails: 3.3 V, 5 V, and 12 V. The module was used to power the sensors (3.3 V), the Arduino (5 V), the 120 mm brushless fan (12 V), etc.

**Figure 5:**
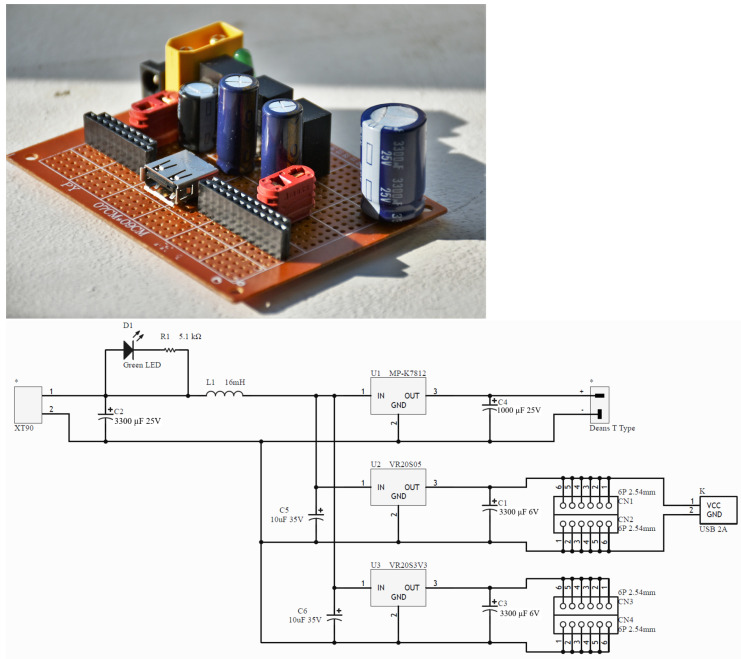
Upper: Multi-rail power supply module. Lower: circuit diagram for power supply. Component Description: XT90: XT90 Input connector to Battery. D1: Green Power Input Indicator LED. R4: 5.1 kΩ Resistor. C1: 3300 µF 6 V Capacitor. C2: 3300 µF 25 V Capacitor. C3: 3300 µF 6 V Capacitor. C4: 1000 µF 25 V Capacitor. C5 and C6: 10 µF 25 V Capacitor. U1: MP-K7812 12 V Switching Regulator. U2: VR20S05 5 V Switching Regulator. VR20S03V3 3.3 V Switching Regulator. CN1–CN4: Output Rails. K: Female USB 2.0 Supply Output.

As seen in Figure 5, it has several connectors that allow for a connection of up to ten 3.3 V devices, eight 5 V devices, and so on. For the 3.3 V rail, the VR20S3V3 regulator was used. It is capable of providing up to 2 A of total current with no need for active cooling at these input voltage levels, especially with the present current consumption, which is estimated to be about 240 mA, for the time being. The 5 V rail uses VR20S05, which also provides up to 2 A, and the expected load consumption is about 150 mA. The 12 V rail features the MP-K7812 regulator, which has a maximum current output of 1 A. The fan is expected to consume 150 mA. All three are switching regulators with efficiencies between 89% and 96%, depending on several factors, such as the input voltage, load current demands, etc. Although the regulators themselves are capable of accepting an input voltage of up to 36 V, the capacitors used in this circuit are the limiting factors since they are rated for an operating voltage of 25 V, which meets the requirements of this project. This was done to optimize volumetric efficiency, as higher voltage capacitors are significantly larger and unnecessary for the current application.

For safety purposes, an emergency stop switch was also installed, to allow anyone to bring the experiment to an immediate stop by cutting off the power to the entire system.

**Figure 6:**
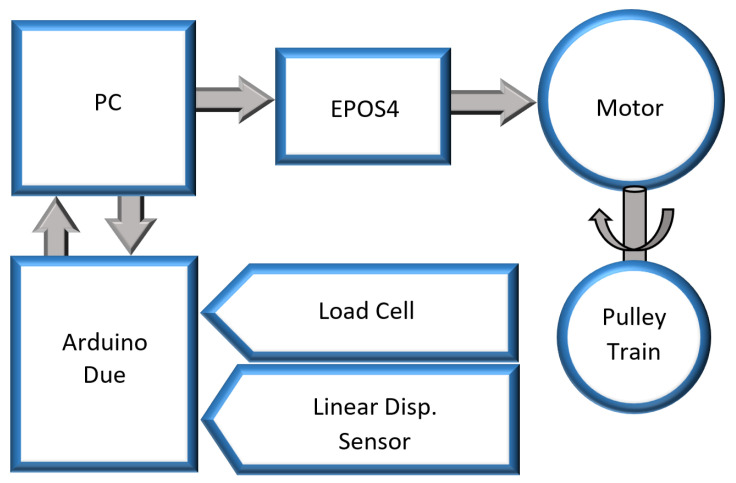
Block diagram of the test bench components.

The overall system block diagram is presented in Figure 6.

## 3. Applications and Tests Conducted

The components described in the previous section allow one to perform numerous experiments by varying the combination of modules used in each configuration. This work will describe a few of the tested configurations in order to determine operating parameters for different parts of the exosuit and to validate the practicality and adaptability of the test bench. This section will describe the experiments conducted and their results will be discussed in the following section.

### 3.1. Tuning the Motor Position Controller

The first test involved mounting the motor on the appropriate support with the corresponding pulleys to be used, albeit without any cables attached. The goal of this procedure was to optimize the control system operating parameters by taking into account the total rotational inertia of the entire transmission system to be used.

**Figure 7:**
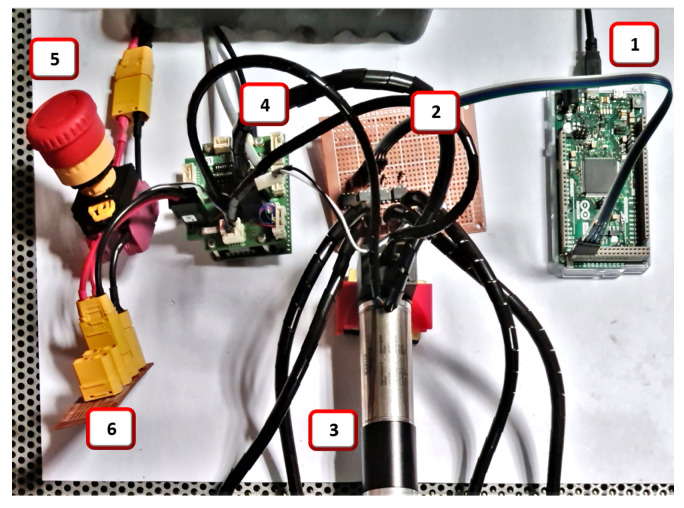
Some modules mounted on the test bench: (1) Arduino DUE, (2) clutch control module, (3) BLDC motor, (4) EPOS4, (5) emergency stop button, and (6) voltage bus module.

This type of test should be performed under a controlled environment with appropriate safety measures in place, thus making it an ideal candidate for the test bench instead of testing it on the exosuit itself. The motor was fed a pulse curve, which moved it to a predefined position and back to the original position. The setup is shown in Figure 7.

### 3.2. Multi-Sensor Validation

To actuate the joints appropriately, the correct estimation of the cable displacement is important. This depends on several factors, such as the cable winding, the anchor points, tension from the cable to the pulley, etc. For these tests, the pulleys have an initial winding that allows them to release the cable if the input curve demands it. In order to have the cables tensioned correctly, cable stops are used, which permit the fine adjustment of the cables coming out from both pulleys in a train, and the motors are made to go to a predefined pretension position so that the cables have appropriate tensioning applied before starting the experiment. When the test is running and the motor is given a position curve, the demanded and obtained displacement values are compared to determine whether any of the potential error sources, such as cable rolling, pulley supports, guides, anchor point friction, etc., cause any significant errors in the test results. This test is conducted with no scale factor applied, as the real-world overall displacement needs to be similar to that encountered in the prototype exosuit for the test to be useful. By sending the input curve, the resulting displacement can be measured or estimated using different types of sensors, and the results are compared to help decide the best combination to meet a given experiment’s needs. The encoder is an indispensable part of every configuration as it provides feedback to the position control algorithm and is used in all tests. The load cell is very good at detecting small changes in the cable tension and estimated displacement. After applying a pretension position to ensure that all of the attached cables are tensioned sufficiently, a tare is applied to the load cell readings to minimize errors and enhance repeatability. The linear displacement sensor has the advantage of being able to measure real cable displacement without being affected by external noise in cable tension variations. It is also used as part of the feedback loop in some tests where the resulting cable extension is important.

### 3.3. Optimizing Clutch Performance

In order to isolate the actuation of the knee joint from the hip and ankle actuation, as described in Section 2.1, electromechanical clutches were used. The clutch control board input was provided by an Arduino and the output was connected to the 24 V electromechanical clutch. The test bench was used to test the clutching performance, in order to adjust the plate distance, test the designed clutch control board performance, etc. This was done by spinning the motor at a constant velocity and then actuating the clutch while measuring the slip (if any), the power consumption at the clutch control board, and the heat dissipated using K-type thermocouples.

### 3.4. Testing Synergy-Based Actuation Methods

Once the test bench was calibrated after conducting the aforementioned tests, the next step involved applying different actuation curves obtained by synergies to the motors in order to evaluate the optimal actuation method for the exosuit. This involved attaching specially designed pulley trains to each motor, with individual diameters determined based on the synergies of the hip and ankle. Then, cables were routed from the respective pulley of each leg segment between the two motors. At the point of intersection, where the cable from each motor meets the pulley and springs, sensors such as load cells and linear displacement sensors were installed to measure the resulting synergy produced.

**Figure 8:**
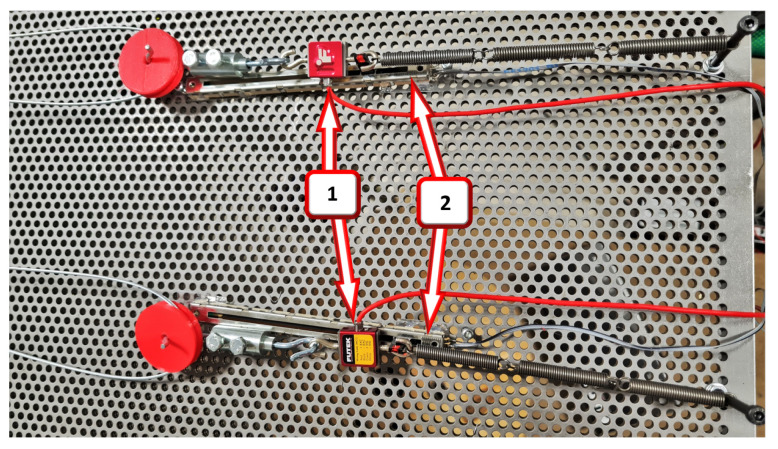
Load cells (1) and linear displacement sensors (2).

The details of the load cells, linear sensors, series springs, anchor points, middle pulleys, and cable stops are depicted in Figure 8. Each sensor pair measures a specific segment: one for the hip and another for the ankle.

## 4. Results

### 4.1. Tuning the Motor Position Controller

**Figure 9:**
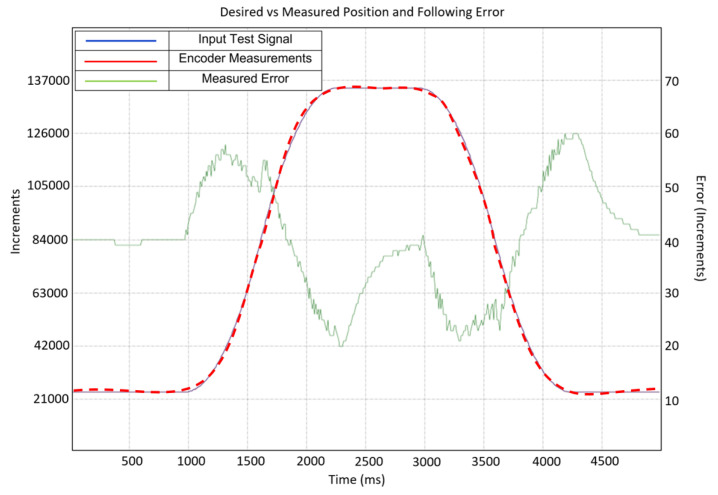
PID tuning desired vs. measured with the following error. Blue: input test. Red: discontinuous, reported position. Green: error.

The graph in Figure 9 shows the input curve sent to the motor (blue), the position measured by the encoder (red), and the following error (green). The motor was configured to move at high velocity and acceleration values of 10,000 rpm and 4300 rpm/s, respectively, to test the motor at high loads with the gearbox and gear reductions, which might be necessary in the final exosuit design, taking into account the reduction in the gearbox and other transmission elements.

As can be seen in Figure 9, the motor follows the position curve with a high level of precision. Even with the aforementioned high velocity and acceleration inputs, the maximum following error was about 60 increments, which corresponds to a resulting position error at the output pulley of about 0.639°, showing that the PID loop is well-tuned. This loop could be tuned further for improved acceleration or a lower error, but for the current requirements of this project, these results are satisfactory and allow for experiments with high levels of precision and repeatability.

### 4.2. Multi-Sensor Validation for Cable Displacement Algorithms

**Figure 10:**
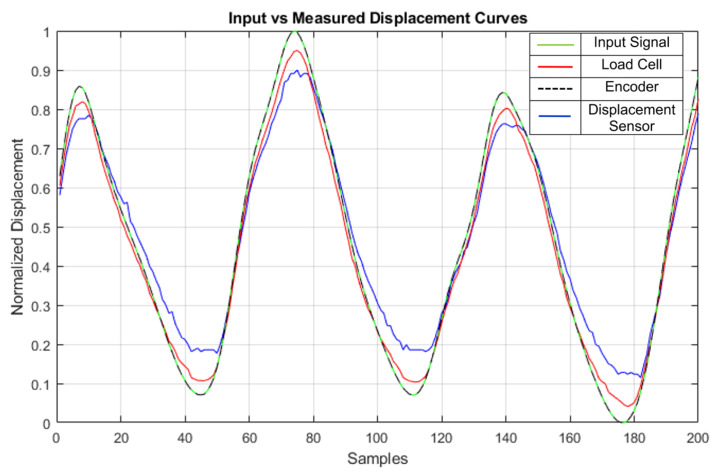
Multi-sensor displacement. Green: input signal. Black dashed: encoder. Red: load cell. Blue: linear displacement sensor comparison.

In one of the tests, the estimated cable displacement was measured by the encoder, load cell, and linear displacement sensor. The results are shown in Figure 10:

As can be observed, the three sensors correctly measured the input synergy curve that was fed to the motor during the experiment, with curves that were very similar to the input signal (green line). The encoder (dashed black line) flawlessly follows the input curve, making it difficult to distinguish in the graph. The cable displacement measured by the load cell (red) using Equation (4) is also very similar to the input curve, showing a slight reduction compared to the input displacement at both peaks. This error could be attributed to limitations in terms of the configurations, types of springs used, and friction. The linear displacement sensor (blue) has more error due to the limited rigidity in the connection between where its shaft connects to the cable. This could be remedied by printing a better clamp, which allows it to be attached firmly to the cable to be measured. It did, however, perform better in other applications with larger cable displacements. The results show that all three sensors are well-calibrated and comply with their roles in the given application, but perhaps the mounting points could be further improved if more accurate results are required for a certain application. As the test bench is modular, the sensors can be added or removed in order to meet the needs of a particular experiment.

**Figure 11:**
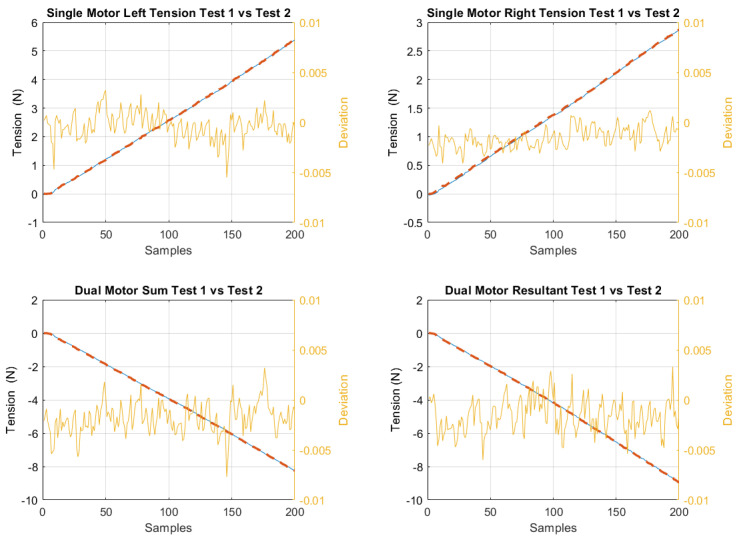
Comparison of cable tensions across four different experiments, each repeated twice. Blue: test 1. Red dashed: test 2. Orange: deviation.

Each test was repeated a couple of times to adjust the system parameters, such as the cable tension, initial positions, etc., until consistent results were obtained for each experiment. When comparing the results of each test, the maximum difference in measurements, such as the resulting cable tension between tests, was calculated to be from 2.3 to 5.4% in preliminary tests, with multiple cables and motors being actuated simultaneously before any adjustments were made, to as low as 0.3–0.48% in tests with cable tension, anchor points, and other system parameters that were tuned carefully, depending on the complexity of the experiment performed. A few of these results can be seen in Figure 11. Two of the graphs involve tests with differently sized pulleys on a single motor, while the other two present the resultant cable tension measurements taken when two motors with pulleys were used to actuate a joint together. These are well within acceptable limits, which would be of the order of 8% and above, and would not be noticeable in the quality of assistance provided by the exosuit.

### 4.3. Optimizing Clutch Performance

Using the test bench, the performance of the clutch control board was evaluated by feeding it an input signal and testing the actuation performance. The resulting clutching performance was improved over a few tests by changing the distance between the plate, which helped the electromagnet hold on to the clutch plate more firmly than before. At 24 V, a continuous current of about 320 mA was measured when the clutch was switched on, which was well within the limits of the control board’s solid-state relays, even taking into account an inductive spike when actuated. When using K-type thermocouples to measure temperatures, the clutches did not cause significant heat dissipation in the solid state relays under load. It was, however, determined that the clutches themselves became slightly warm under heavy continuous loads, reaching temperatures of up to 63 °C, which led to the decision to include an efficient BLDC fan in the final design of the exosuit to provide active cooling.

### 4.4. Testing Synergy-Based Actuation Methods

A preliminary analysis of synergies was conducted to obtain results of two segments: the hip and the ankle.

**Figure 12:**
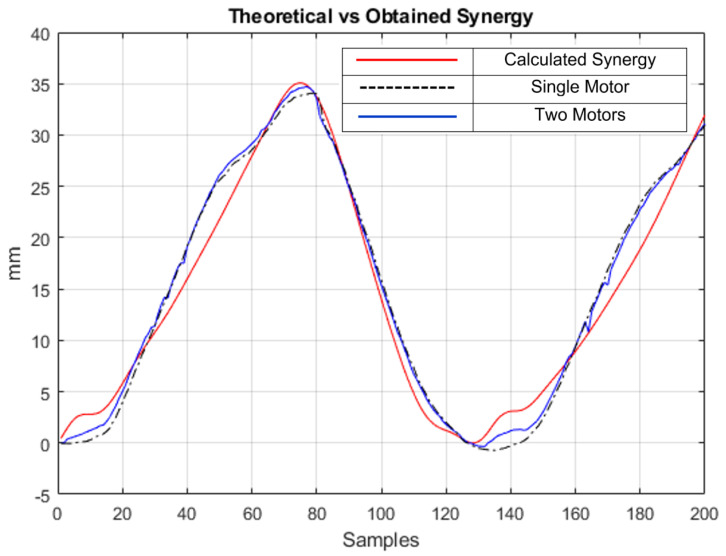
Obtained synergy in terms of cable displacement. Red: theoretically calculated value. Black dashed: single motor. Blue: resultant with two motors.

The theoretically calculated synergy curve is shown in red on Figure 12. In the same figure, the dashed black line is the resulting displacement of one single motor, whereas the blue line is the resulting displacement of the synergy between two motors. As is abundantly clear, the two curves are very similar to each other, with the single motor configuration only losing some detail at a few points in the curve in samples 5 and 135, approximately, when compared to the input curve and the version with two motors. The resulting dual motor displacement of 34.7 mm means that the actual expected displacement would be about 138.8 mm once the scaling factor is taken into account. The single motor displacement of 34.0 mm means that the actual expected displacement would be about 136.0 mm after scaling. Compared to the calculated result of about 140 mm, the results are really close (99.14% with two motors vs. 97.14% with one motor) and minor deviations from the expected values could be primarily attributed to inconsistencies in the test setup, such as printed pulley dimensions differing from the desired CAD design due to contractions, cable self-winding, inconsistent cable tension, etc., apart from the scaling error, thus validating the mathematical model based on synergies, and showing that actuation using just a single motor for all three segments of the leg can be implemented on the exosuit without losing much quality in the final gait assistance, as long as appropriately sized pulleys are used to actuate each segment. The main goals of this experiment are to prove that the design approach based on synergies is indeed possible, and may bring about many benefits to exosuit designers, reducing the overall system weight and price, and making it more accessible to more people. Regardless of whether the experiment is conducted on a person wearing an exosuit or on a test bench, the conclusion does not depend on measurement errors. When following the authors’ approach, decreasing the number of actuators will not necessarily imply relevant errors in the actuation, leading to a more efficient design approach. This result also shows that the real-world system implemented on the test bench and the algorithms discussed in this paper are configured correctly, giving outputs similar to the mathematically predicted system response.

## 5. Conclusions

Looking at the results, the test bench is capable of testing different types of actuation strategies, which eventually helped in the development of a prototype exosuit based on postural synergies, significantly reducing the number of actuators required. In practice, it has shown that the transmission system can follow the curves of the input signal correctly, or with acceptable errors, in most cases, with exceptional repeatability between test results, in addition to proving the great advantage of the synergy-based approach when compared to traditional design approaches for lower limb assistance.

It has indeed helped greatly in quantifying the real-world difference in actuation between one and two motors and proving the concept of synergies, as evidenced by the results. The results show that a single motor can reproduce the required gait assistance for the hip–ankle and knee, given an appropriately designed pulley train without losing much quality in the resulting gait assistance, leading to results really close to the mathematically calculated estimates. This can help in designing exosuits that are much more economical by reducing the number of actuators used. The minuscule quality loss corresponds to the highly accumulated variance in kinematic variables related to gait, such as instantaneous cable extensions.

The bench was indispensable in the optimization of system parameters for the control of the motors (iterative improvements of PID parameters, anchor points, clutch plate distances), actuation strategies using one or two motors, component dimensioning (for the cables), etc.

Given the dimensions of the test bench, it is capable of reproducing actuation strategies with zero to minimal input signal scaling, having used a scaling factor of just 1:3 (at most) for the tests conducted thus far. Tests of the hip and knee were conducted with no scaling factor applied, and a small scaling factor was used in the ankle test with displacement and springs. When weights are used instead of springs, the system can perform tests with zero scaling applied, as would be required with the exosuit.

Compared to the other test benches for exosuit design and testing, this bench is able to perform the same simple, single-motor experiments mentioned in those prototypes, as well as other, more complex experiments involving more than one motor, as well as multiple pulley trains involving cable-driven actuation, such as the test involving synergies detailed in this article. The modular nature of the bench allows the majority of the components to be reused between experiments, saving the time needed to design specific hardware for each test setup. The voltage module allows all components used in testing to be powered from it directly and it still has free pins for future expansion. The clutch control module allows up to four clutches to be used in the experiments and is shown to be very efficient, as evidenced by the results. The code on the Arduino allows the attached sensors to be polled independently by the script running on the PC, allowing only the sensors necessary for a particular experiment to be read without the need to reprogram the Arduino (by just using a different script on the host PC). The motor mount supports pulleys of different sizes, facilitating the testing of pulley trains of different dimensions, without the need to rebuild the entire system. Testing the actuation system for different subjects is as simple as printing a new pulley with the correct diameters, as calculated by the mathematical models, and mounting it on the same bench with a recalculated input trajectory if necessary, allowing for extremely quick testing of different actuation strategies. This applies to the test bench itself as well as the exosuit based on this design.

Different types of control algorithms can also be implemented on the bench to facilitate comparisons between torque- vs. position-based models, among others. Compared to other test benches, which are purposely built for particular experiments, the proposed design is much more flexible as it allows for testing different configurations of drive mechanisms, anchor points, transmission systems, etc. Since the test bench has several holes distributed in a uniform manner across its entirety, it is easy to add more components in order to perform more complex future experiments. For instance, in the modeling of muscular viscoelasticity, additional motors could be added instead of springs to simulate nonlinear load behavior. This could be used to simulate effects such as spasticity, weak muscles, gait abnormalities, etc., to simulate different types of patients, with the aim of designing a control scheme that is capable of adapting to these irregularities.

Considering all of the results above, the test bench has proven its usefulness as an invaluable tool for the testing of synergy-based exosuit actuation systems, saving time, and helping to minimize prototyping and construction costs. As far as improvements go, one useful inclusion would be the addition of a protective metal case with a tempered glass viewing panel, akin to those used for CNC machining, which would allow this test bench to be used for much tougher testing, without compromising safety. Currently, motors are not made to run at full power for these safety reasons. Another application would involve stress-testing the electronics used, which would help prevent any harm in case a component such as a capacitor fails unexpectedly. A case involving a side panel window would enable safe testing of such experiments while allowing the user to observe the experiment in progress.

## Data Availability

Data sharing is not applicable to this article.
